# How One of Our Editors Is Regarded by the Lay Press

**Published:** 1898-08

**Authors:** 


### HOW ONE OF OUR EDITORS IS REGARDED BY THE LAY PRESS.

Dr. Rush Shippen Huidekoper, who knows more about horses and dogs and cats than any other man that ever attained social recognition in Philadelphia and Newport and at the New York horse-show, is one of the many heroes who have declared their intention to shed their blood, if necessary, in defence of American honor.

Dr. Huidekoper has gone to the front. He weighs something less than three hundred pounds, and is the most active man of his weight and years that I know.

Dr. Huidekoper is probably the most valuable man that chappydom has contributed to the war. Not only is he a fighter, but he is an expert surgeon. When he isn't killing Spaniards he can be taking care of the men that the Spaniards wound, and when he isn't doing either of these things he can be looking after the health of the horses.

He won his laurels as a cross-country rider years ago in and about Philadelphia, and since he took up his residence in New York he has enjoyed the unique distinction of being the only horse-doctor in the Four Hundred.

At Newport Dr. Huidekoper is both loved and feared by that extraordinary species of the genus homo, the Newport cabby. He is loved by this class for his knowledge of the horse, and he is feared by them on account of the remarkable things which he does.

One summer at Newport Mrs. Fred. Neilson had a dog that had something the matter with one of its eyes. Dr. Huidekoper was sent for, and made a diagnosis of the malady of the unfortunate beast. He declared that a surgical operation was necessary; that one of the eyes of the dog had to be removed and be replaced with a glass eye. The proposition delighted Mrs. Neilson. She consented at once. Her coachman told other coachmen of the remarkable surgical experiment that was to be made, and these coachmen told the cabbies, until there wasn't a horse-driver in the whole of the city by the sea who did not know what was going on, and who did not ask permission to see Dr. Huidekoper operate. The doctor graciously acceded to the request of the coachmen and the cabbies to be present. They watched him take out the diseased eye of the dog and replace it with a glass eye. They were loud in their expressions of amazement at his skill.

The doctor received their compliments and commendations placidly. He said that it was nothing. He declared that both the animal eye and the human eye could be removed at will and without pain. Then he coolly dropped one of his own eyes into his hand and replaced it in the presence of the gaping congregation. Ever since then the cabbies at Newport have looked at him with awe. They regard him as a man with the evil eye. They do not know that the eye he dropped into his hand and replaced is a glass eye.

If the doctor can work the same trick with the same success on the Spanish, it is probable that he will go through the war unscathed.

---

[For the JOURNAL.]

### POOR PUSSY.

POOR Pussy, weak and unprotected;
Unmindful of the ways of man;
Now thou art old, therefore neglected
By those who do and can.

What if thine orb has dimmed by age,
  And cold and stiff thy joints;
Does man successful lose the page,
  Away from victory points?

Ah, pussy, would that man could know
  The secret of thy senses,
For worth can gather what it sow;
  All things have recompenses.

Your steps are slow, poor pussy, now;
  Your midnight wail is ended,
And many a time I wished, I vow,
  Your feet had from me wended.

But now man's cruel hand of fate
  Decrees thy worth is waning,
And rains upon thy lowly state
  Insults, without his gaining.

Compassion is the fruit of love,
  And age needs much caressing;
I would that I could live and move
  As thou, no faults possessing.

Ah, pussy, mindful of thy fate,
  Can man do less than wonder;
Oft seeming small is truly great;
  The seeming great asunder.

Ah, pussy, recollection's train
  Begets a common ending
For I and thou; but what thou gain
  Has been within the sending.

And what has been is all in all,
  The past, and now, and after,
While I wait the welcome call,
  To see my Lord and Master.

—F. B. BRUBAKER, M.D.

MIFFLINBURG, PA.

